# Supplementary material for: Automated model building and protein identification in cryo-EM maps
Source: Nature. 2024 Feb 26;628(8007):450–7. doi: 10.1038/s41586-024-07215-4 (PMC11006616; doi:10.1038/s41586-024-07215-4)
Supplement: Supplementary file 1 — List of all EMDB/PDB entries in the first test set of 177 structures (entries in bold are also part of the second test set of 27 structures). [file 41586_2024_7215_MOESM1_ESM.pdf]

nature portfolio

https://doi.org/10.1038/s41586-024-07215-4

Supplementary information

Automated model building and protein identification in cryo-EM maps

In the format provided by the
authors and unedited

Automated model building and protein identification in cryo-EM maps

Kiarash Jamali [1*], Lukas Käll [2], Rui Zhang [3],
Alan Brown [4], Dari Kimanius [1*] & Sjors H.W. Scheres [1*]

[1] MRC Laboratory of Molecular Biology,
Cambridge, UK
[2] Science for Life Laboratory, KTH Royal Institute of Technology,
Stockholm, Sweden
[3] Washington University in St. Louis,
St. Louis, MO, USA
[4] Blavatnik Institute, Harvard Medical School,
Boston, MA, USA
[*] Correspondence to {kjamali,dari,scheres}@mrc-lmb.cam.ac.uk

List of all EMDB/PDB entries in the first test set of 177 structures.

Entries in bold are also part of the second test set of 27 structures.

EMDB entry	PDB code	Reported resolution
emd-26595	7ulw	3.10
emd-26608	7ums	3.50
emd-26616	7un8	3.30
emd-26617	7un9	3.30
emd-26701	7uqx	3.30
emd-26702	7uqy	3.00
emd-26712	7urg	3.46
emd-26732	7usc	3.00
emd-26733	7usd	3.00
emd-26734	7use	3.00

EMDB entry	PDB code	Reported resolution
emd-26742	7usx	3.09
emd-26743	7usy	3.54
emd-26754	7ut4	3.90
emd-26756	7ut6	1.91
emd-26757	7ut7	1.91
emd-26760	7ut8	2.43
emd-26763	7ut9	2.44
emd-26770	7uth	3.90
emd-26782	7utn	2.74
emd-26801	7uur	1.67
emd-26838	7uwq	3.05
emd-26841	7uws	3.47
emd-26858	7uxe	3.38
emd-26886	7uze	2.40
emd-26916	7uzq	2.17
**emd-26917**	**7uzs**	**2.20**
**emd-26948**	**7v0q**	**2.50**
emd-26949	7v0s	2.50
**emd-33187**	**7xgr**	**2.60**
emd-33192	7xha	3.35
emd-33193	7xhb	3.33
**emd-33233**	**7xjp**	**2.71**
emd-33242	7xk3	3.10
**emd-33243**	**7xk4**	**3.10**
emd-33244	7xk5	3.10
emd-33245	7xk6	3.00
emd-33305	7xmu	2.30
**emd-33306**	**7xmv**	**2.60**
emd-33309	7xn3	2.90
emd-33310	7xn4	3.35
emd-33311	7xn5	3.18
emd-33312	7xn6	3.45
**emd-33331**	**7xnz**	**3.60**
emd-33348	7xoh	3.60
emd-33430	7xsp	2.89
emd-33431	7xsq	2.88
emd-33432	7xsr	2.97
emd-33433	7xss	3.20

EMDB entry	PDB code	Reported resolution
emd-33439	7xt4	3.08
emd-33528	7xzi	2.77
emd-33529	7xzj	2.97
emd-33540	7y0d	3.10
emd-33621	7y5n	3.45
emd-33676	7y80	2.71
emd-33677	7y81	2.54
emd-33678	7y82	2.83
emd-33719	7yat	2.20
emd-33802	7yfz	3.19
emd-33807	7yg4	3.10
emd-33853	7yi8	2.70
emd-33854	7yi9	2.60
**emd-33861**	**7yim**	**2.60**
emd-33955	7yn9	3.53
emd-33956	7yna	3.64
emd-33957	7ynb	3.46
emd-33958	7ync	3.14
emd-33959	7ynd	3.29
**emd-34017**	**7ypx**	**3.12**
emd-34023	7yqc	2.82
emd-34024	7yqd	3.40
emd-14714	7zgx	2.88
**emd-14716**	**7zh0**	**3.20**
**emd-14725**	**7zh6**	**3.67**
emd-14733	7zhj	3.53
emd-14774	7zl1	2.50
emd-14777	7zl4	3.45
emd-14842	7zny	3.26
emd-14847	7zol	3.03
emd-14848	7zoq	3.20
emd-14869	7zqb	3.88
emd-14873	7zqp	3.60
emd-15039	7zzq	2.60
emd-15043	8a00	2.60
emd-15044	8a01	3.20
emd-15046	8a03	3.20
**emd-15047**	**8a04**	**3.20**

EMDB entry	PDB code	Reported resolution
emd-15048	8a05	3.40
**emd-15220**	**8a7d**	**3.06**
emd-15285	8a9l	2.20
emd-15361	8ade	2.78
emd-15370	8adu	3.24
emd-15378	8ae1	3.25
emd-15540	8ane	3.20
**emd-15560**	**8ap7**	**2.70**
**emd-15561**	**8ap8**	**3.70**
emd-15635	8at6	3.70
emd-15646	8atd	3.10
emd-15673	8aur	3.47
emd-15684	8avv	3.40
emd-15685	8avw	3.62
**emd-15686**	**8avx**	**3.50**
emd-15690	8aw3	3.60
emd-15785	8b0j	3.99
emd-15930	8b9f	3.93
**emd-15960**	**8bc2**	**2.60**
emd-16183	8bqe	3.50
emd-15949	8bsh	3.80
emd-16437	8c5i	3.15
emd-16511	8c9m	3.20
**emd-26973**	**8csw**	**2.50**
emd-26974	8csx	2.40
emd-26976	8csz	3.20
emd-26978	8ct2	3.10
emd-26993	8ctk	3.52
emd-26994	8ctl	3.10
emd-27020	8cvx	3.50
emd-27021	8cvy	3.60
**emd-27022**	**8cvz**	**3.52**
emd-27104	8d0b	3.43
emd-27138	8d1v	2.82
emd-27173	8d43	2.88
emd-27175	8d45	2.62
emd-27252	8d8n	3.60
emd-27253	8d8o	3.35

EMDB entry	PDB code	Reported resolution
emd-27320	8dc2	2.99
emd-27421	8dgc	3.40
**emd-27431**	**8dh7**	**2.99**
emd-27542	8dmk	3.70
**emd-27574**	**8dnm**	**2.76**
emd-27639	8dpn	2.49
emd-27645	8dq0	3.74
emd-27656	8dql	3.00
emd-27661	8dqv	1.52
**emd-27755**	**8dwi**	**3.40**
emd-27758	8dws	3.73
**emd-27760**	**8dwu**	**3.40**
emd-27761	8dwv	3.60
emd-27795	8dze	2.99
emd-27796	8dzf	3.69
emd-27797	8dzg	3.10
emd-27875	8e40	3.57
**emd-27899**	**8e50**	**3.67**
emd-27903	8e55	3.85
emd-27936	8e76	2.51
emd-27937	8e78	2.77
emd-27945	8e8o	2.77
emd-28064	8eex	2.95
emd-28065	8eey	2.53
emd-28080	8efd	3.80
**emd-28081**	**8efe**	**3.80**
emd-28129	8egs	3.92
emd-28233	8em4	2.83
emd-28241	8em7	2.97
emd-28244	8emc	3.60
emd-28248	8emh	3.63
**emd-28637**	**8evu**	**2.58**
emd-28641	8ew3	2.65
emd-28660	8exr	3.80
emd-28666	8ey2	3.50
emd-28781	8f0u	3.10
emd-28866	8f5o	3.50
emd-28867	8f5p	3.40

EMDB entry	PDB code	Reported resolution
emd-29289	8fm9	3.20
**emd-29290**	**8fma**	**3.10**
emd-29323	8fnt	2.52
emd-29326	8fnu	2.50
emd-29327	8fnv	2.11
emd-34158	8gna	2.80
emd-34270	8gu6	3.10
emd-31702	8h03	2.80
emd-31704	8h05	3.40
emd-34430	8h1p	3.48
emd-34678	8hdr	3.66
emd-34679	8hds	3.57
emd-34738	8hgg	3.64
emd-34963	8hr8	3.30
emd-34965	8hra	3.76
